# Supplementary figures and images for: CEP290 is essential for the initiation of ciliary transition zone assembly
Source: PLoS Biol. 2020 Dec 28;18(12):e3001034. doi: 10.1371/journal.pbio.3001034 (PMC7793253; doi:10.1371/journal.pbio.3001034)

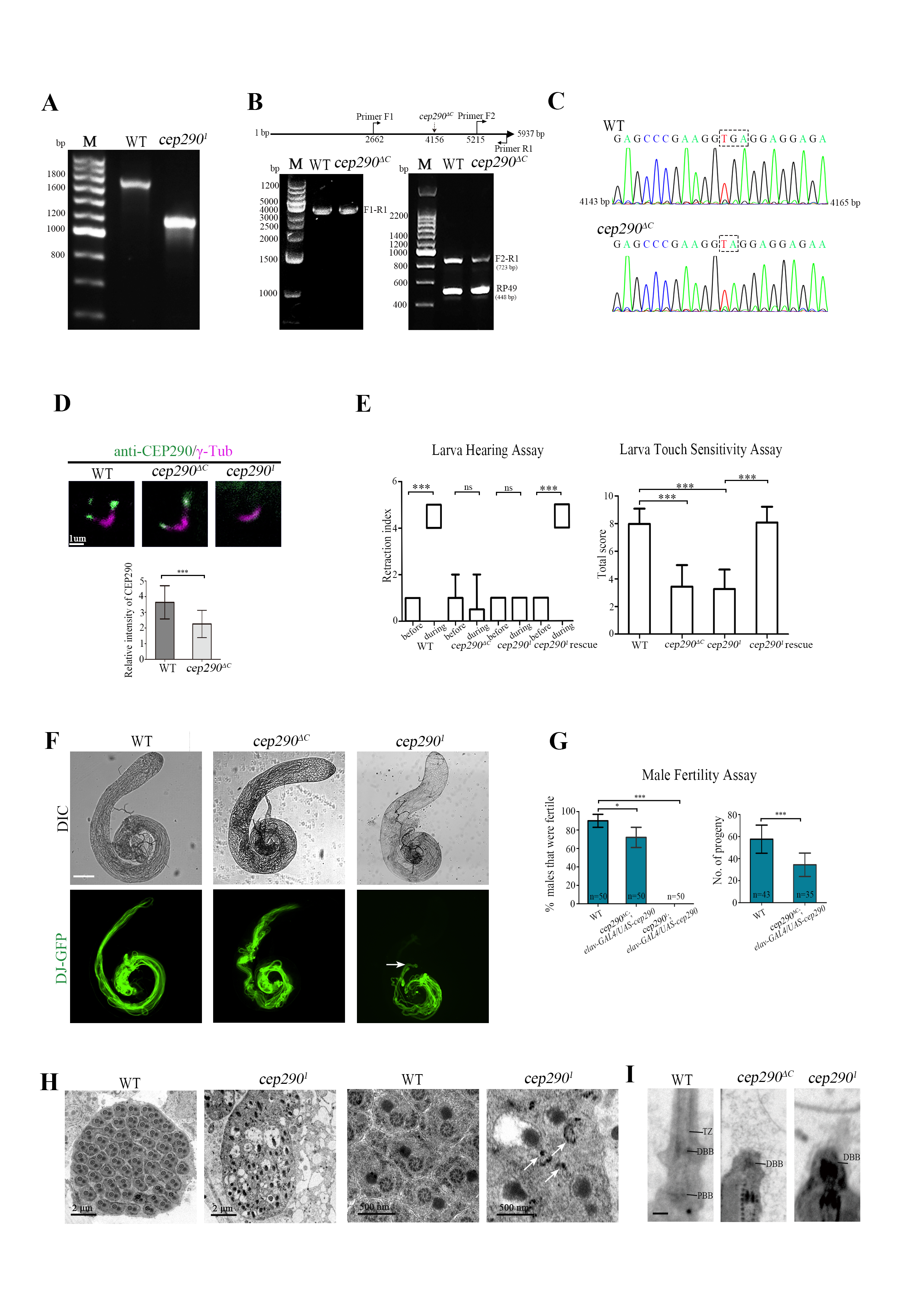

Supplement: S1 Fig — (A) Genotyping of cep2901 mutants by PCR of CEP290 fragment spanning the deletion region using whole fly genomic DNA. The amplification products were 1,754 bp long for w1118 and 929 bp long for cep2901 mutants. (B) RT-PCR analysis of the splice isoforms and transcription level of cep290 gene in cep290ΔC mutants. Amplification of the C-terminal half of cep290 CDS (2662–5937) did not show other splice forms caused by the deletion mutation. Semiquantitative RT-PCR analysis of the transcription level of cep290 CDS using primer F2 and R1 showed that the transcription level was reduced in cep290ΔC mutant, probably due to nonsense-mediated mRNA decay. Housekeeping gene rp49 was used as the internal control. (C) cDNA sequencing verification of cep290ΔC mutant. (D) Upper panel: Immunofluorescence staining with an anti-CEP290 N (aa292-541) antibody confirmed that the CEP290 N-terminal signal was reduced in cep290ΔC mutants but completely lost in cep2901 mutants. The corresponding relative fluorescence intensity was quantitatively displayed in lower panel. The bars and error bars represent the means and SDs, respectively. n = 50 centrioles over 5 flies. Scale bars, 1 μm. (E) Cilia-related behavior analysis of cep290 mutants. Both cep290ΔC and cep2901 mutant flies have severe defects in hearing (left panel) and touch sensitivity (right panel). Expression of CEP290 rescued these defects. For the hearing assay, 5 larvae as a group, and at least 5 groups of flies were tested. For the touch sensitivity assay, n = 50. (F) Testes of WT flies and cep290 mutants. The mitochondrial protein DJ was used to label sperm cysts. Compared to those in WT flies and cep290ΔC mutants, sperm cysts in cep2901 mutants were severely defective in elongation. The arrow indicates that the cysts failed to elongate. Bar, 200 μm. (G) Male fertility assay in WT flies and cep290 mutants. We first rescued the severely uncoordinated phenotype of cep290 mutants by expressing CEP290 driven by elav-GAL4. The perce [file pbio.3001034.s001.tif]

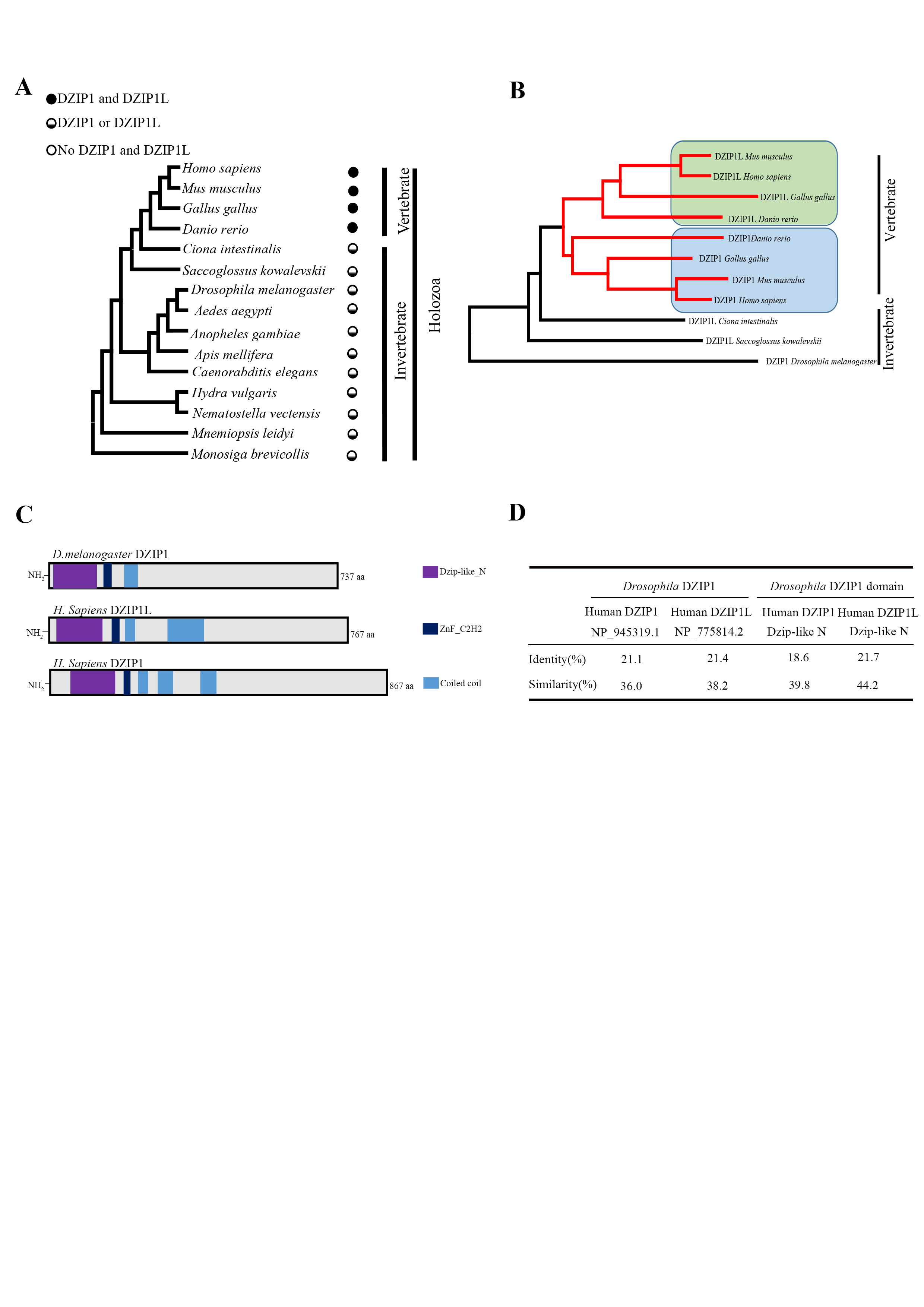

Supplement: S2 Fig — (A) DZIP1 is evolutionarily conserved in Holozoans. There is only 1 DZIP1 gene in invertebrates, while there are 2 genes in vertebrates, DZIP1 and DZIP1L, possibly because of gene duplication. (B) Phylogenetic tree of DZIP1. Drosophila DZIP1 is orthologous to both DZIP1 and DZIP1L. (C) Schematic representation of the protein structure of Drosophila DZIP1 and human DZIP1 and DZIP1L. All share highly conserved domain that includes Dzip_like-N and ZnF_C2H2. (D) Table showing the sequence similarity and identity between Drosophila DZIP1 and human DZIP1 and DZIP1L. (TIF) [file pbio.3001034.s002.tif]

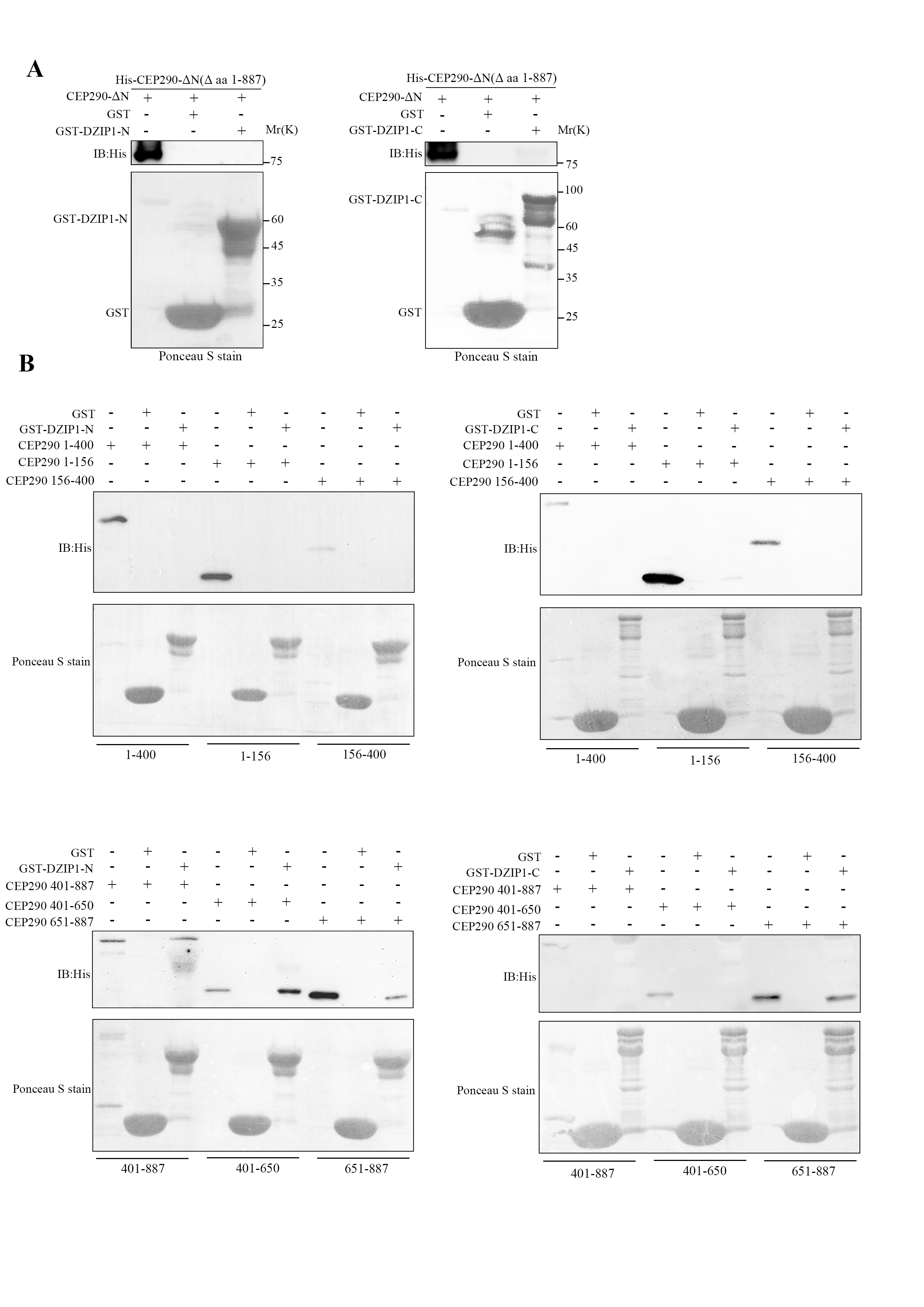

Supplement: S3 Fig — (A) DZIP1 did not interact with CEP290-ΔN (Δ aa 1–887) in the GST pull-down assay. (B) The GST pull-down assay was used to narrow down the interaction region between CEP290 and DZIP1. DZIP1-N (aa 1–293) interacts with both CEP290 N (aa 401–650) and CEP290 N (aa 651–887), and DZIP1-C (aa 294–737) interacts with CEP290 N (aa 651–887). Uncropped immunoblots can be found in S2 Raw Image. CEP290, centrosomal protein 290; DZIP1, DAZ interacting zinc finger protein 1; GST, glutathione S-transferase. (TIF) [file pbio.3001034.s003.tif]

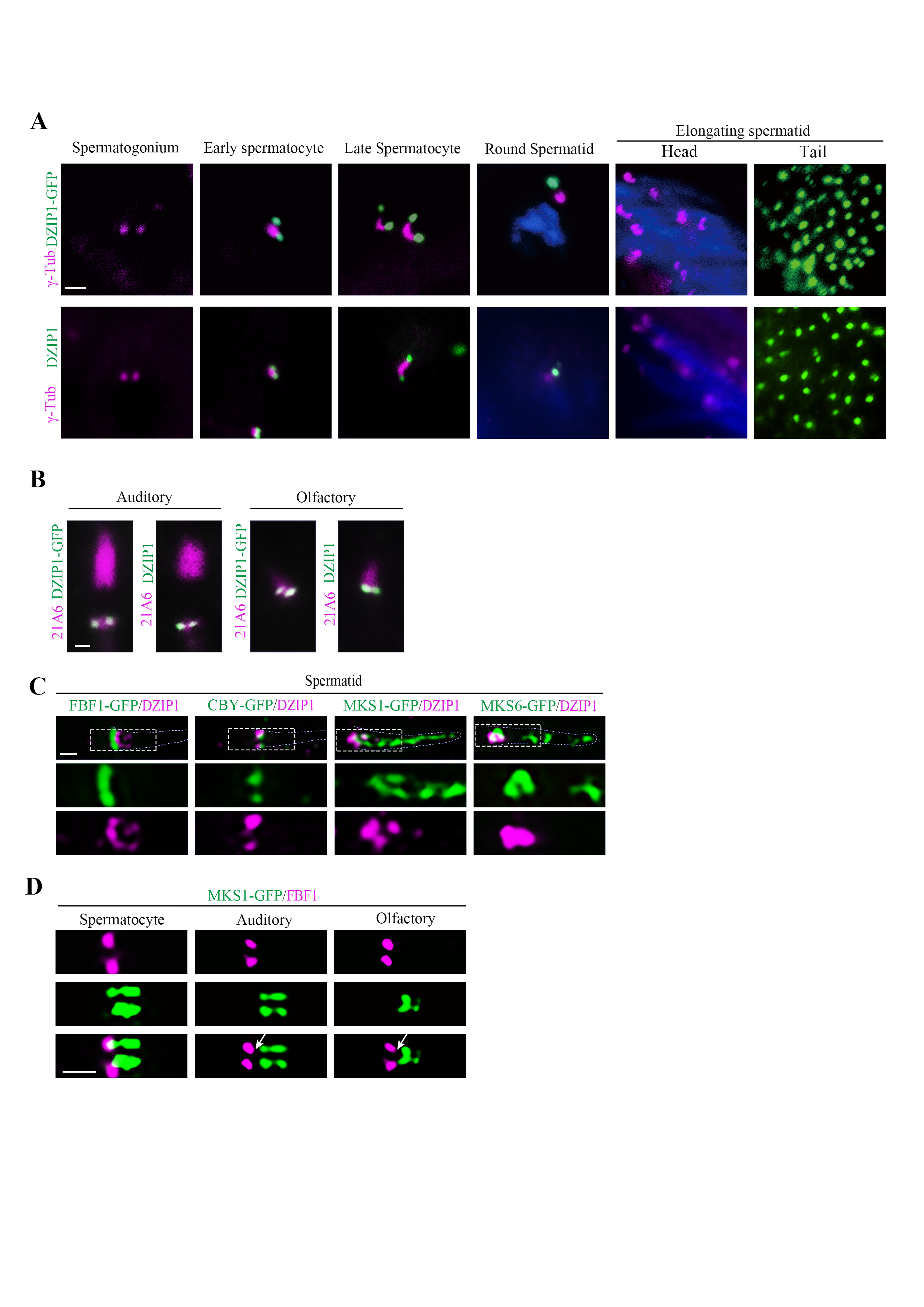

Supplement: S4 Fig — (A) Subcellular localization of DZIP1 during spermatogenesis in Drosophila. Upper panel: DZIP1-GFP; lower panel: anti-DZIP1. DZIP1-GFP and anti-DZIP1 staining showed similar localization patterns. No DZIP1 was present on centrioles in spermatogonia. DZIP1 began to appear on the tips of centrioles in early spermatocytes. In round spermatids, DZIP1 migrated together with the ring centriole to the tip of flagella. The centrioles/BBs were labeled with γ-Tubulin (red). Bar, 2 μm. (B) DZIP1 localized to the ciliary base in both auditory and olfactory cilia. Ciliary bases were labeled with 21A6 (red). Anti-DZIP1 staining showed a localization pattern similar to that of DZIP1-GFP. Bar, 1 μm. (C) 3D-SIM images of DZIP1 localization in spermatids, DZIP1 colocalized with CBY at the ring centriole, but did not extend like MKS1. Bar, 500 nm. (D) 3D-SIM images of spatial relationship between TF marker FBF1 and TZ core protein MKS1 in various types of cilia. Notably, there is a gap (arrows) between FBF1 and MKS in sensory cilia but not in spermatocyte cilia. Bar, 500 nm. 3D-SIM, three-dimensional structured illumination microscopy; BB, basal body; CBY, Chibby; DZIP1, DAZ interacting zinc finger protein 1; FBF1, Fas binding factor 1; GFP, green fluorescent protein; MKS, Meckel–Gruber syndrome; MKS1, Meckel syndrome type 1; TF, transition fiber; TZ, transition zone. (TIF) [file pbio.3001034.s004.tif]

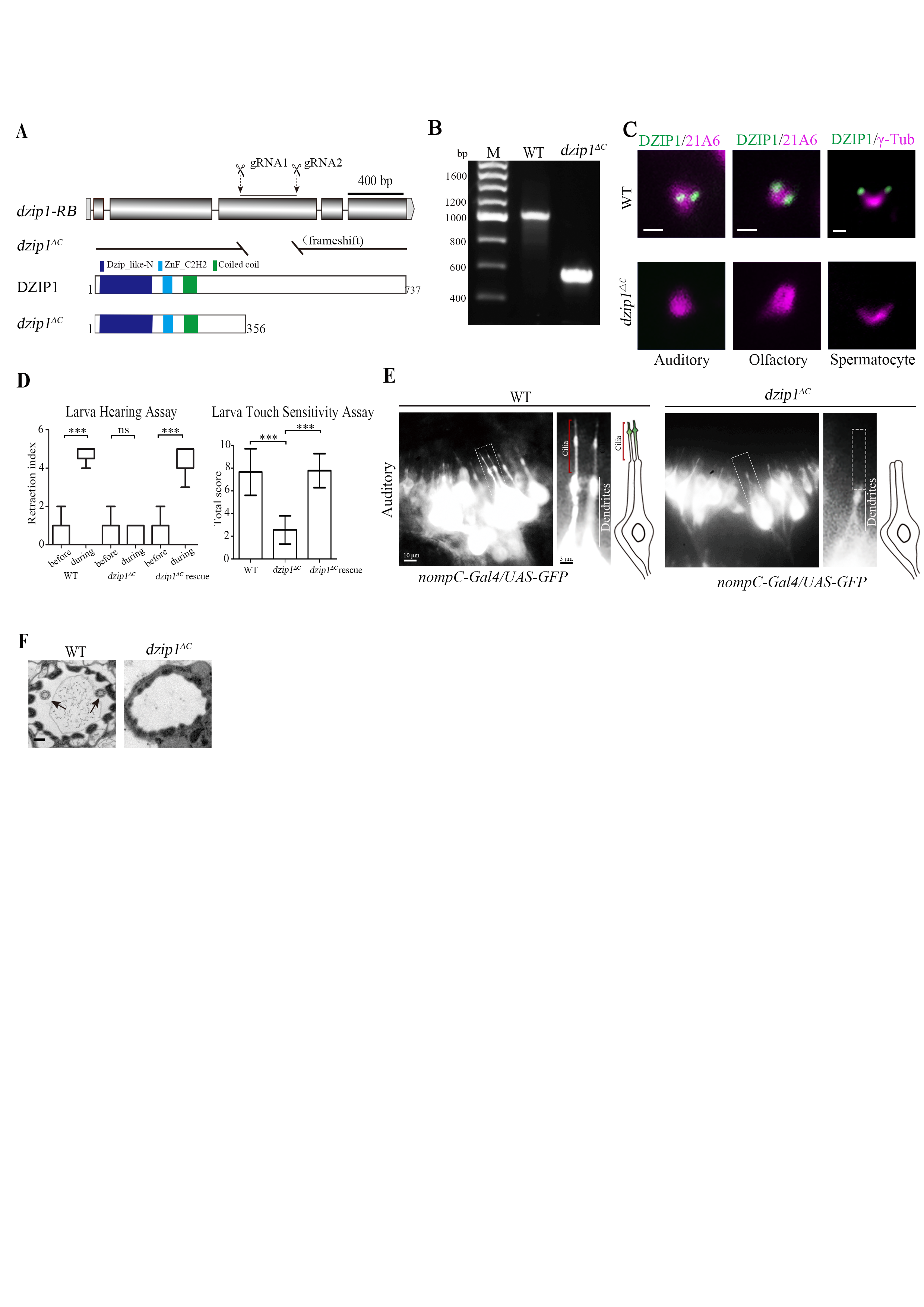

Supplement: S5 Fig — (A) Generation of the dzip1 deletion mutant. Schematic of the dzip1 gene. The gRNA target sites are represented by scissors. dzip1ΔC has a deletion of cDNA from nt 1,069 to 1,529, resulting in a frameshift and a premature stop codon and leading to the loss of the C-terminus. (B) Genotyping of the dzip1 mutant. The sizes of the PCR products are as follows: 1,070 bp in w1118 flies and 609 bp in dzip1ΔC flies. (C) Anti-DZIP1 staining confirmed that DZIP1, at least the C-terminus of DZIP1, was completely lost in dzip1ΔC mutants. Bars, 1 μm. (D) dzip1ΔC mutant flies showed defects in hearing and touch sensitivity, but expression of DZIP1-GFP driven by its own promoter rescued these defects. The retraction index is shown as the median and interquartile range. Numerical data can be found in the file S1 Data. The bars and error bars represent the means and SDs, respectively. n = 50. (E) nompC-Gal4; UAS-GFP was used to mark auditory cilia. Cilia completely disappeared in dzip1ΔC mutants. (F) EM images of cross sections of cilia showing that no axonemes existed in dzip1ΔC mutants. The black arrows indicate ciliary axonemes in WT flies. Bars, 200 nm. DZIP1, DAZ interacting zinc finger protein 1; EM, electron microscope; GFP, green fluorescent protein; gRNA, guide RNA. (TIF) [file pbio.3001034.s005.tif]

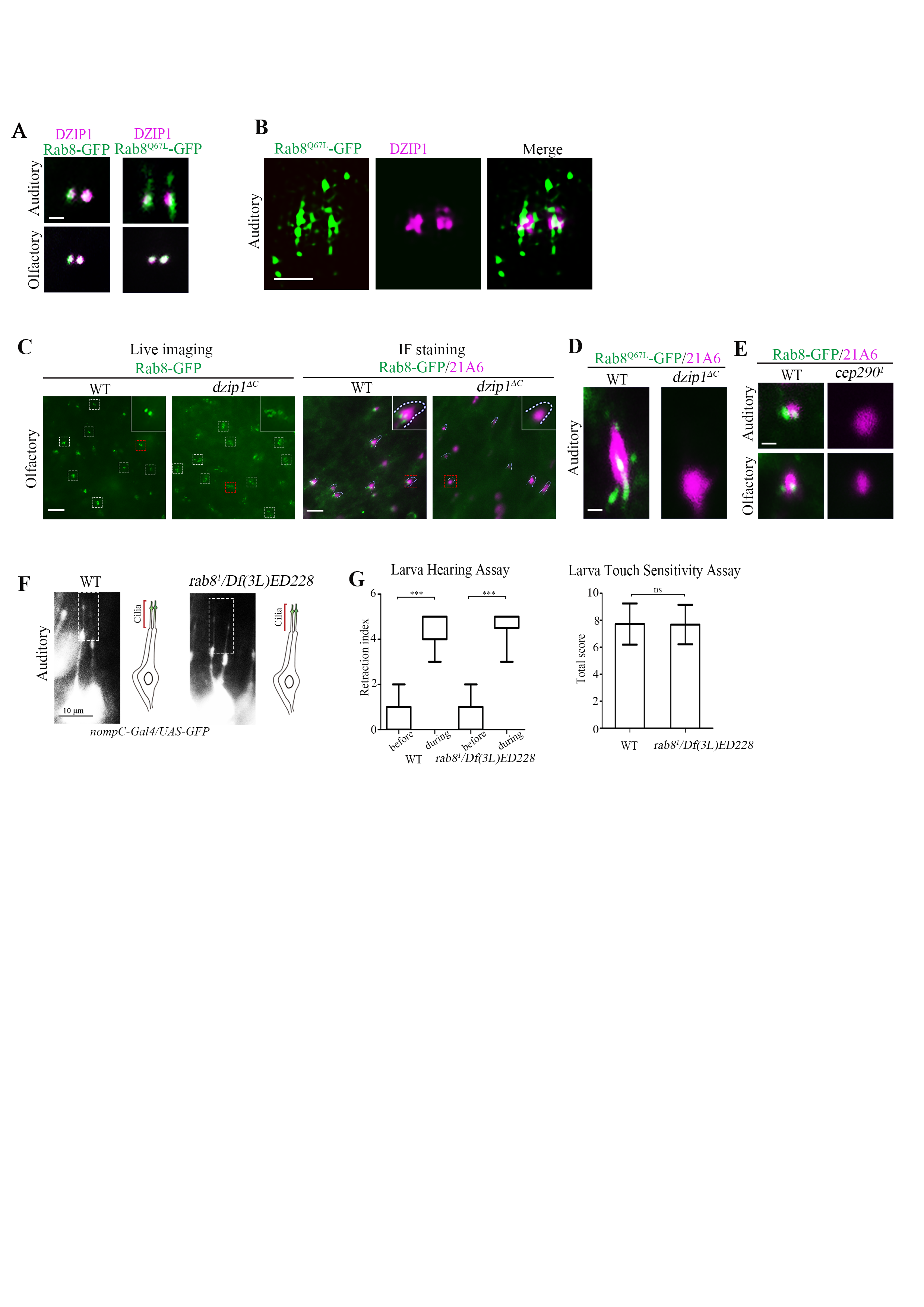

Supplement: S6 Fig — (A) Rab8-GFP and Rab8Q67L-GFP colocalized with DZIP1 at the ciliary base in auditory cilia and olfactory cilia. Notably, Rab8Q67L-GFP was frequently observed in cilia. Bar, 1 μm. (B) 3D-SIM images of the colocalization of Rab8Q67L-GFP and DZIP1 at the base of auditory cilia. Rab8 Q67L-GFP surrounded DZIP1 at TZ and expanded into cilia. Bar, 1 μm. (C) DZIP1 was critical for Rab8-GFP localization at the ciliary base of olfactory cilia. Live imaging showed that Rab8-GFP exhibited a clear 2-dot pattern at the ciliary base in WT flies, but the signal was dispersed in dzip1 mutants. Interestingly, these dispersed signals completely disappeared in our immunofluorescence assay, most likely due to fixation. Bars, 5 μm. (D) DZIP1 was required for Rab8 Q67L-GFP localization at the ciliary base of auditory cilia. Bar, 1 μm. (E) Rab8-GFP was completely lost from the ciliary base of both auditory cilia and olfactory cilia in cep2901 mutants, indicating that CEP290 is essential for Rab8 localization. Bar, 1 μm. (F) Cilium morphology was normal in rab81 mutants. (G) rab8 mutants exhibited normal hearing responses and normal touch sensitivity. Numerical data can be found in the file S1 Data. 3D-SIM, three-dimensional structured illumination microscopy; DZIP1, DAZ interacting zinc finger protein 1; GFP, green fluorescent protein; TZ, transition zone; WT, wild-type. (TIF) [file pbio.3001034.s006.tif]

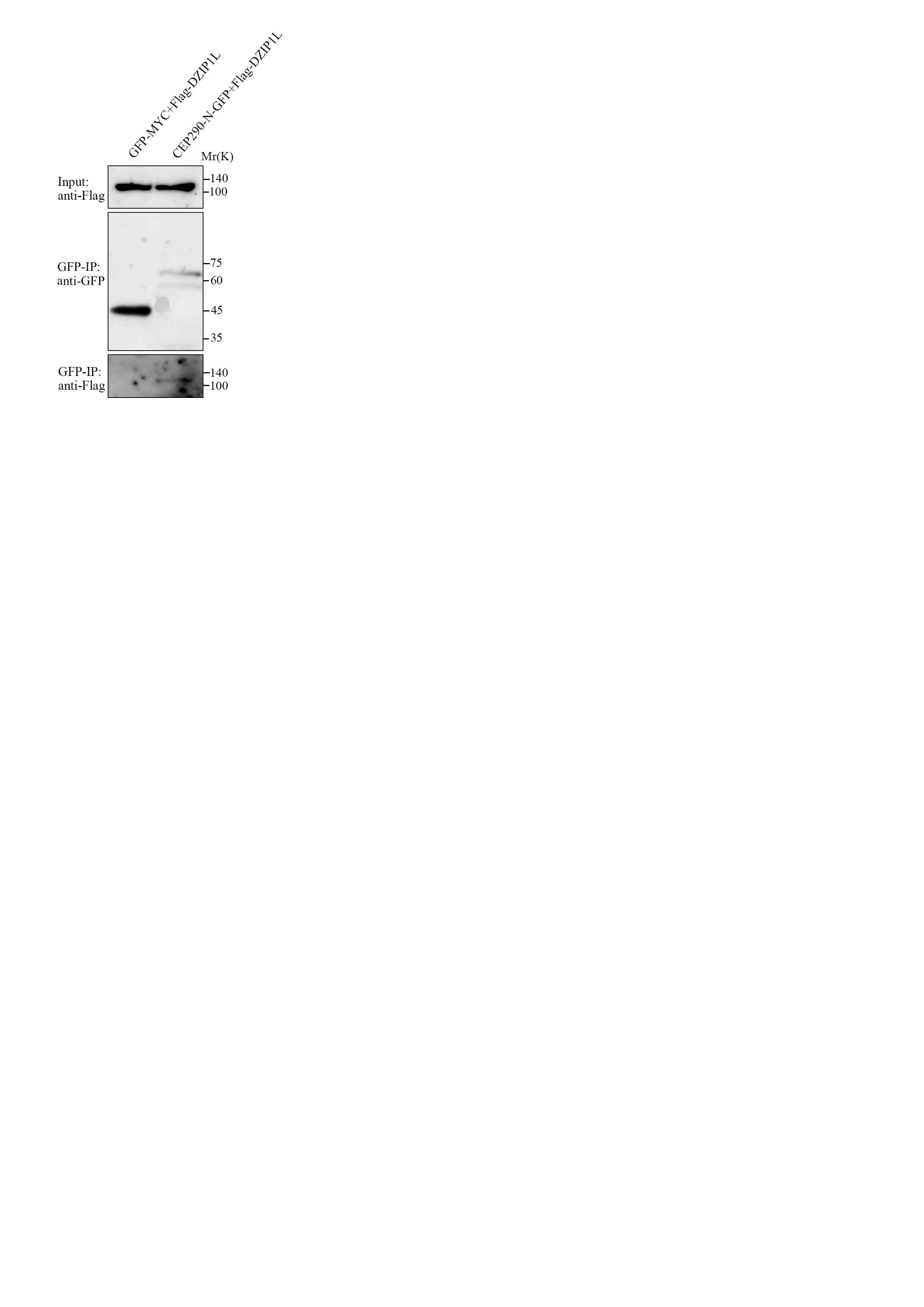

Supplement: S7 Fig — The interaction between human CEP290 and DZIP1L was analyzed using an immunoprecipitation assay. Human CEP290-N-GFP and Flag-DZIP1L were transiently transfected into HEK293 cells; 48 h later, cells were lysed and subjected to Co-IP using GFP-trap beads. Uncropped immunoblots can be found in S2 Raw Image. CEP290, centrosomal protein 290; Co-IP, coimmunoprecipitation; GFP, green fluorescent protein. (TIF) [file pbio.3001034.s007.tif]

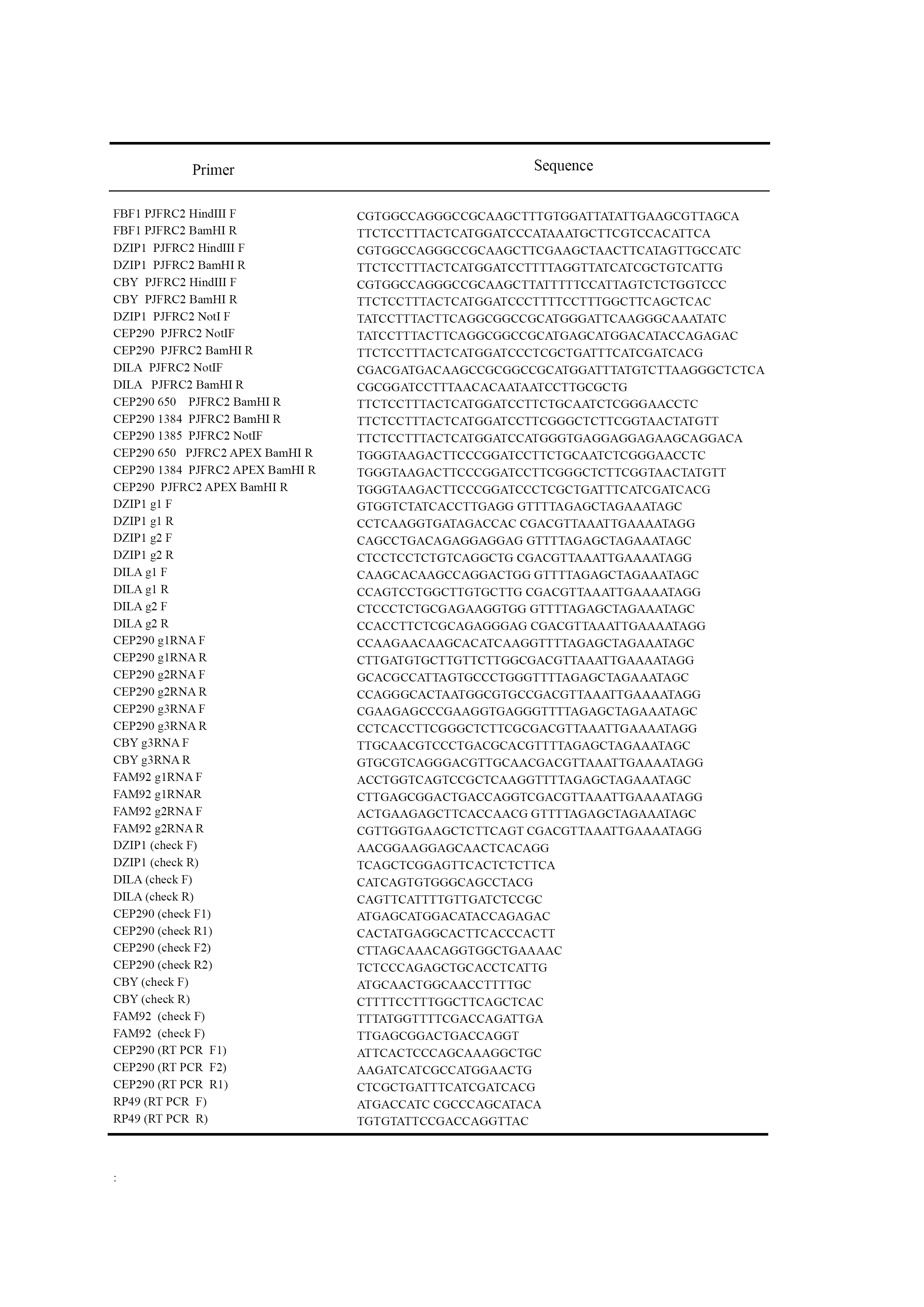

Supplement: S1 Table — (TIF) [file pbio.3001034.s008.tif]

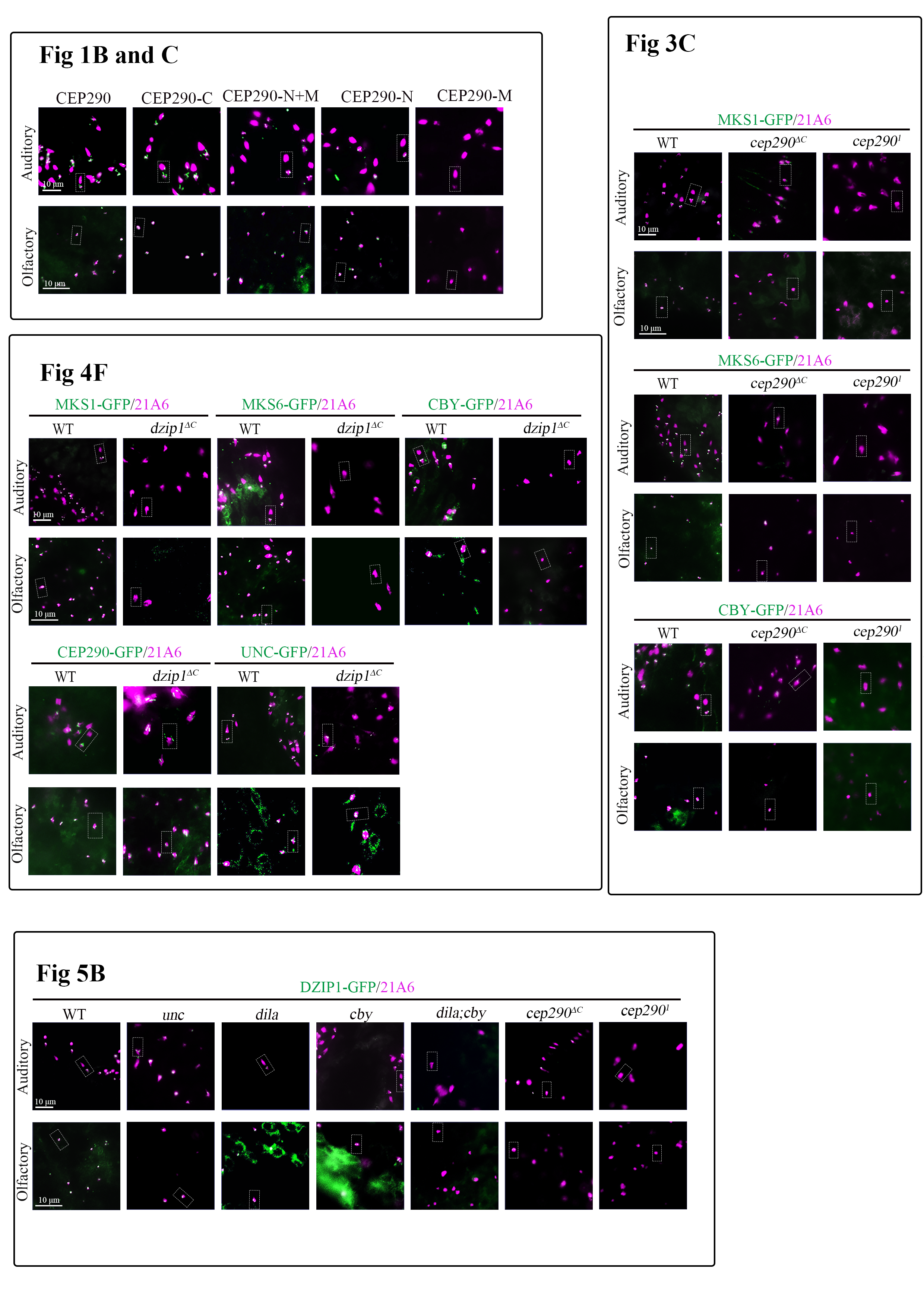

Supplement: S1 Raw Image — (TIF) [file pbio.3001034.s010.tif]

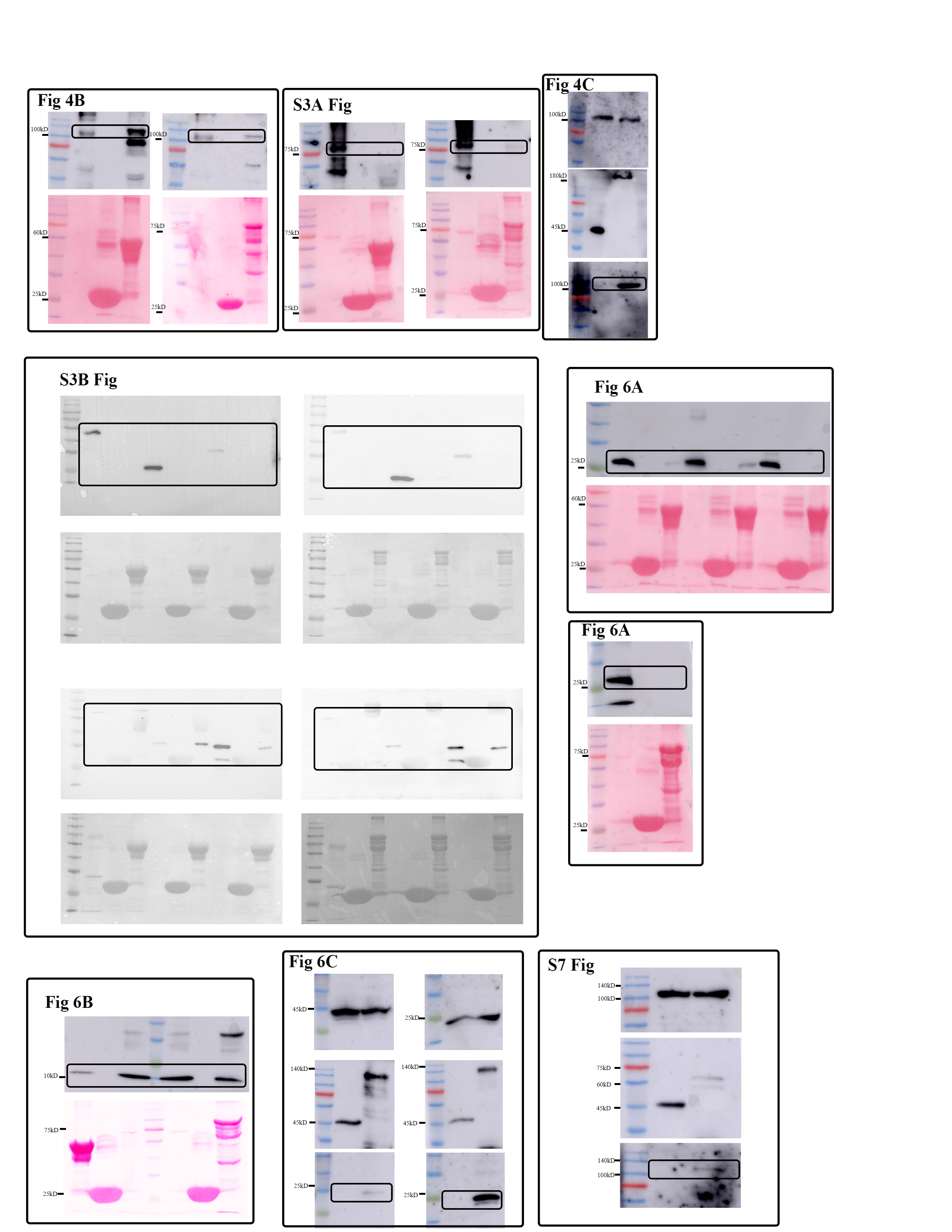

Supplement: S2 Raw Image — (TIF) [file pbio.3001034.s011.tif]
