# Supplementary material for: CEP290 is essential for the initiation of ciliary transition zone assembly
Source: PLoS Biol. 2020 Dec 28;18(12):e3001034. doi: 10.1371/journal.pbio.3001034 (PMC7793253; doi:10.1371/journal.pbio.3001034)
Supplement: Attachment — Submitted filename: Response to Reviewers final(1).docx [file pbio.3001034.s012.docx]

We thank all reviewers for their critical comments, which help us ultimately strengthen the manuscript.

**Reviewer #1:**

**Major points:**

**Question 1.** While a variety of experimental approaches are employed to demonstrate binding between Cep290-N and Dzip1, a relatively large fragment of Cep290 is used (aa 1-650). This region has previously been shown to have various important functional properties (homodimerization; binding to Cp110, Pcm1, and Nphp2; membrane binding via a conserved amphipathic helix), and thus it is possible that the various phenotypic defects attributed to loss of Cep290-N are not due to loss of Dzip1 binding per se but due to disruption of one or more of these other functional elements (in fact the phenotypes seen in Cep290-l mutants that are not present in Cep290-deltaC could be due to any elements within aa 1-1385). This concern is partly mitigated by the similarity of phenotypes seen in Cep290-l and Dzip1 mutants, but it would be helpful if the authors can narrow Cep290's Dzip1 binding region more precisely and analyze ciliary phenotypes when Dzip1 binding is more selectively disrupted (e.g. by

rescue of Cep290-l with such a targeted Cep290 mutant). At a minimum this issue should be discussed in more detail in the manuscript.

We agree with the reviewer that the various defects in *cep290^1^* mutant can’t be attributed solely to DZIP1. CEP290 plays multiple roles in ciliogenesis through a variety of binding partners (not DZIP1 exclusively). This complexity is exemplified by various functional studies revealing that it is not only required for the initiation of ciliogenesis, but also involved in the subsequent TZ assembly and axoneme formation.

In this manuscript, we showed that *cep290^1^* mutant and *dzip1* mutant have similar defects in the initiation of ciliogenesis and TZ assembly in *Drosophila*. We found that CEP290 is required for the proper basal body localization of DZIP1, its N-terminal interacts with DZIP1, and expression of CEP290 N (aa 1-650) alone restores the localization of DZIP1 and enables DZIP1 dependent initiation of ciliogenesis and TZ assembly. Therefore, we propose that CEP290 mediates the initiation of ciliogenesis and TZ assembly mainly via DZIP1. However, although expression of CEP290 N could rescue defects in the initiation of ciliogenesis and TZ assembly, it could not fully rescue defects in TZ assembly and axoneme formation, indicating that CEP290 acts through other interaction partners independent of DZIP1. As the Reviewer suggested, we discussed this issue in our revised version.

For the function of CEP290 N-terminal in the initiation of ciliogenesis and TZ assembly, we could not rule out possible contributions of other known CEP290 binding factors. As the Reviewer suggested, we have tried to narrow down the precise interaction site between CEP290 N-terminus and DZIP1. As showed in Supplementary Fig. 3B, we have found that CEP290 N (aa 400-887) interacts with DZIP1. However, a further precise disruption of CEP290/DZIP1 interaction through targeted mutagenesis as the reviewer suggested is unlikely to be instructive, since this is still a large fragment, we have uncovered a myriad of mutual interactions between these two proteins. For example, DZIP1-N (aa 1-293) interacts with both CEP290 (aa 401-650) and CEP290 (aa 651-887), DZIP1-C (294-737) interacts with CEP290 (aa 651-887). For the reason mentioned above, we are unable to conduct the precise rescue experiment as suggested by the Reviewer.

**Question 2.** A key point in the manuscript is that the N-terminus of Cep290 has key roles in transition zone formation. This conclusion relies on the more severe phenotypes observed in the Cep290-l mutant than the Cep290-deltaC mutant. However, the predicted frameshift produced by the Cep290-deltaC allele is not experimentally verified, and the possibility of alternative splice forms and/or altered transcript stability is not considered. Given the key conclusions based on this mutant, it would be very helpful if the authors could better characterize precisely what form(s) of Cep290 are expressed in this mutant and at what levels relative to wildtype.

To address this question, we raised an anti-CEP290 antibody against CEP290 N-terminal (aa292-541). Using immunofluorescence assay, we demonstrated that CEP290 N signal was completely lost in *cep290^1^* mutant, but it still existed in *cep290^ΔC^* mutants, even though the signal intensity was significantly reduced (Supplementary Fig. 1D). This result indicated that CEP290 N-terminal do exist in *cep290^ΔC^* mutant. Unfortunately, the antibody doesn’t work in western blot assay, we can’t use it to determine if CEP290 is indeed truncated at the precise CRISPR engineered site in *cep290^ΔC^* mutant.

Nevertheless, we analyzed the transcripts of *cep290* in *cep290^ΔC^* mutants. The CDS of *Drosophila* *cep290* gene is 5937 bp long. There is a one bp deletion (c.4156delG) in *cep290^ΔC^* mutant. As the full length CDS is too large to amplify, we amplified the C-terminal half of *cep290* CDS (2662-5937) in both wild type and *cep290^ΔC^* mutant. No alternative splice forms were detected (Supplementary Fig. 1B). To determine whether the transcription level was affected. Semi-quantitative RT-PCR was used to amplify a shorter cDNA fragment. As showed in Supplementary Fig. 1B, the transcription level was indeed reduced in *cep290^ΔC^* mutant, probably due to nonsense-mediated mRNA decay.

**Minor points:**

**1.** Is there any evidence from the authors or others indicating that the reported connection between Cep290 and Dzip1 might be conserved in other species? Similarly, can the pathogenic features of any disease-associated CEP290 mutants potentially be attributed to disruption of the Cep290-Dzip1 interaction? Experiments examining these points may be beyond the immediate scope of this paper but could make for an impactful addition; at minimum further discussion of these points would be helpful.

To our knowledge, there is no report that CEP290 is functionally linked to DZIP1/DZIP1L in other species. We have unpublished preliminary data showing that human CEP290 interacts with DZIP1L (see below), suggesting that the interaction should be evolutionarily conserved.

It has been reported that mammalian DZIP1/DZIP1L interacts with Rab8 and CBY (Reference 36, 37, 40), and DZIP1L is critical for ciliary bud formation in mammal (Reference 36). Thus, combined with our experimental data, we speculate that a critical function of DZIP1/DZIP1L in early ciliary membrane formation is likely conserved in mammals. Although no reports connect CEP290 to DZIP1 in mammals yet, it has been reported that CEP290 is involved in ciliary vesicle formation (Reference 55); and JSRD fibroblasts showed defects in ciliary membrane formation (Reference 47*)*; and cilia are completely lost in *Cep290* KO cell lines (*Elif Nur Firat-Karalar, Turk J Biol 2018; 42: 371-381*)*.* Therefore, it is possible that defects in early ciliary membrane formation in *cep290* patients may related to DZIP1/DZIP1L. As the reviewer suggested, we have included further discussions in revised version.

**2.** There are some confusingly worded sentences and grammatical errors throughout the text (particularly in the last section of the Results).

We apologize for our writing. We revised the manuscript, rewrote the last section of results, and have the manuscript proof-read by commercial language-editing service.

**Reviewer #2:**

Whereas the manuscript conclusions on Cep290 are well supported by the observations, the role of Rab8 in TZ assembly is not clearly demonstrated and the authors' conclusion is largely overstated. In particular, the phenotype of the rab8 mutant is weakly documented and does not support their conclusion. To be able to conclude on Rab8 function in TZ assembly in Drosophila, the authors should at least address the following points:

-show the rescue of the phenotype of cby; rab8 double mutant by Rab8-GFP and, even more demonstrating, not by Rab8T22N.

-the sole phenotype described for the cby; rab8 double mutant is a reduction in MKS1-GFP at the TZ? Is this the only phenotype observed for this double mutant? Is there any consequence on behavioral assays? What is the testis phenotype of the rab8 mutant alone and the double cby; rab8 mutant? Is it consistent with a function in TZ assembly? My opinion is that the results on rab8 are much too preliminary to support a role of Rab8 in TZ assembly and actually reduce the impact of the other conclusions that are very interesting.

We thank the reviewer for raising these critical questions.

The ciliary defect in *rab8* single mutant is indeed very weak. The morphology of cilia in auditory neurons of pupa looked normal, no defects were observed in larval hearing and touch sensitivity, and no obvious defects were observed in sperm flagella. *rab8* mutant die at the pharate pupal stage (*West et al., J cell Biol. 2015; 208(7): 931-947*), so we were unable to analysis its role in adult fly’s behavior.

However, we did observe that a small proportion of spermatocyte centriole pairs have elongated CG6652-GFP signal in every *rab8* mutant fly we checked (see below). Elongated CG6652 signal is attributed to loss of the ciliary cap/TZ. So, Rab8 is indeed required for ciliogenesis, even though the effect is relatively weak, probably due to the redundancy with other factors, such as Rab10. Notably, similar phenotype was also observed in *rab11* single mutants (see below), supporting that the Rab11-Rab8 cascade does participate in ciliogenesis in *Drosophila*.

Our *dzip1* mutants have much more severely defective phenotypes than *cby* or *fam92* single mutants. Ciliogenesis is completely blocked in *dzip1* mutants, but it is partially affected in *cby* or *fam92* single mutants. These results suggest that defects caused by deletion of DZIP1 cannot be attributed to CBY-FAM92 pathway only. We hypothesis that multiple binding factors of DZIP1 may jointly contribute to ciliogenesis. To test our hypothesis, we investigated whether there is a genetic interaction between two binding factors of DZIP1, CBY and RAB8. Interestingly, when we combined *cby* and *rab8* mutants, we observed that MKS signal was decreased significantly in *cby; rab8* double mutant, compared with *cby* or *rab8* single mutants (Fig. 6E), supporting our hypothesis that DZIP1 recruits multiple independent components to initiate cilligenesis.

In the revised version, we added new data showing that the percentage of centrioles with abnormally elongated CG6652 signal in spermatocytes increased significantly in *cby; rab8* double mutant, compared with *cby* or *rab8* single mutants (Fig. 6F).

Furthermore, as CBY and FAM92 were reported to function in a same pathway (*Lapart et al. Elife 2019; 8: e49307*), we investigate whether deletion of *rab8* could aggravate the phenotype of *fam92* mutant. Indeed, as expected, the percentage of centrioles with abnormally elongated CG6652 signal in spermatocytes increased significantly in *rab8; fam92* double mutant. All these results strongly support that RAB8 and CBY-FMA92 module jointly regulate ciliogenesis.

We have attempted to achieve the rescue experiments the reviewer suggested, by constructing genotypes as *cby; rab8*; *MKS-GFP* or *CG6652-GFP*; *Bam-Gal4*; *UASp-YFP-Rab8.* Flies with the desired genotypes died before their pupal stage, even with repeated attempts. However, overexpression of FAM92 did rescue the phenotype of *rab8; fam92* double mutant (Fig. 6F), which supported our genetic interaction analysis.

**Major points:**

-The authors nicely show that basal bodies (BB) in cep290 mutants do not build any TZ and are, at best, just apposed to the plasma membrane. This result was already proposed by Jana et al., 2018 using RNAi. However, it is not clear why the authors' conclude that the basal bodies are docked: in particular do the authors observe a thickening of the plasma membrane and connections between the membrane and the BB, as described by Gottardo et al, 2018?

From our TEM analysis, we observed that basal bodies are attached/very close to the plasma membrane in early spermatocytes of *cep290^1^* mutant, similar to what *Jana et al., 2018* has proposed. However, technically, it is very difficult to determine whether basal bodies are docked or just apposed to the plasma membrane. We thank the reviewer for raising this question, we have revised our descriptions accordingly.

*Gottardo et al.* reported that thin fibres mediate the early interaction between the basal body and the plasma membrane. However, we have not observed these structures in our mutants. Considering that such thin fibers are structures with unknown composition, even hardly detectable in WT flies, we are not confident to draw the conclusion whether basal bodies are not docked in our mutant. More knowledge about thin fibres or advanced EM techniques are needed to address this question systematically.

How many centrioles were observed by EM? Are they all apposed to the plasma membrane ?

For EM of *cep290^1^* mutant, we cut two testes from two flies, dozens of centrioles were observed, but only 7 larger centrioles show the longitudinal section, and 4 of them are attached/close to the plasma membrane. For centrioles that are not docked to the membrane, there are several possibilities: 1. they might be in the stage before docking; 2. they may have defects in docking; 3. they might be disassociated with the membrane after docking.

On Figure 2G : one of the two basal body does not seem to be docked to the plasma membrane: can this be explained by the level of the section? Do the authors have other sections of this same centriole?

Yes, this is indeed caused by the level of section, please see other sections as provided below.

-The authors analyze the distribution of different Cep290 truncated fragments fused to GFP. This analysis is apparently done in WT conditions where the endogenous protein is present, what happens in cep2901 rescue conditions? Could the difference in the distribution of Cep290 truncated fragments just reveal differences in competition behavior between the truncated and Wt protein?

As shown in figures above, similar to what we observed in WT flies, CEP290, CEP290-N and CEP290-C are all located to the base of auditory cilia and shows different localization pattern in *cep290^1^* mutant. CEP290-GFP labels the TZ, showing a rod-like shape, whereas CEP290-N shows a small dot. Surprisingly, CEP290-C also shows a long rod-like shape. Because CEP290-C couldn’t rescue defects in the TZ assembly and axoneme formation of *cep290^1^* mutant, the signal should not be the TZ. Currently, we do not know the exact nature of CEP290-C localizing structure in *cep290^1^* mutant.

In spermatocytes, similar to what we observed in WT flies, both CEP290 and CEP290-N localize to the tip of spermatocyte centriole in *cep290^1^* mutant. Notably, CEP290-C-GFP, like CG6652-GFP, localizes to the abnormal elongated microtubules.

All the above results suggest that the cilia base localization of CEP290-N and CEP290-C is independent of WT CEP290 protein. Therefore, it is unlikely that the difference in the distribution of CEP290 truncated fragments is caused by differential competitions between the truncated and WT protein.

Minor points:

Overall the IF images of the sensory cilia are of poor resolution. In particular the images of sensory neurons in 1B, 1C, 4F, 5B and D.

The immunofluorescence staining of cilia in sensory neurons of *Drosophila* is hard. We have tried our best to get high-quality images and replaced some of them.

Figure 1. The authors only show one example of the distribution of the different Cep290-GFP fragments: is the distribution homogenous between centrioles? Please quantify the length on several centrioles to show the distribution.

As shown in representative figures provided below, the distribution of CEP290 fragments is homogenous.

As suggested by the reviewer, we have quantitated the signal length of different fragments and plotted the new data in Fig. 1E.

Figure 2C: Quantify the fluorescence intensity in cep290deltaC mutant compared to WT: is there any effect of deleting only the C-terminus, as the flies are hypofertile, and taking into account that mecH alleles show defects in TZ integrity in the testes (Basiri et al., 2014).

We quantified the fluorescence intensity as suggested by the reviewer. Indeed, compared to WT, the signal intensities of MKS1, MKS6 and CBY were significantly reduced in *cep290^ΔC^* mutants, but completely lost in *cep290^1^* mutant. The new representative images and quantitative data were provided in Fig. 2C.

Figure 4D: it was previously shown in Jana et al, 2018, that Cep290 radially extends from the center to the periphery of the TZ, depending on whether GFP is fused to the N-ter or C-ter of the protein. This should be taken into account in the interpretation of the high resolution imaging observations.

We appreciate the suggestion and have discussed accordingly.

Page 6: the authors propose that expressing the N-ter-Cep290 fragment has a dominant negative effect : are all transgenes inserted on the same platform as this is not explained in the methods section? Thus, in all the experiments based on the expression of these truncated forms, could there be an effect of the expression levels?

To ensure that CEP290 and its truncations were expressed at a comparable level, all these transgenes were inserted o the same integration platform (the attP site of 25C6 locus on chromosome 2), and they were driven by the same ubiquitin promoter. Therefore, it is unlikely that the different defective phenotypes with expressing various CEP290 truncations are due to their different expression levels.

Corresponding information has been added to methods section.

Page 7: The authors propose that the C-ter and N-ter domains have an inhibitory effect on their localization and cite Drivas et al. to support their hypothesis. However this latter article shows that overexpressing the N or C-ter domain of human CEP290 leads to increased ciliogenesis in cells. This is completely different to what the authors observe here in their manuscript and is misleading.

We deleted this sentence.

Figure 7 and discussion: there is an apparent confusion in the text and in the figure between what the authors call dense fibers on page 20, which in the referred publications correspond to lateral connections of the proximal (daughter) centriole and the dendrite membrane and what is called "thin fibers" described in Gottardo et al. 2018. These are completely different structures that likely have completely different roles and Figure 7 must be corrected to "thin fibers" as described in Gottardo et al. The EM images of this manuscript do not show that these connections are still present in cep290 mutants. In addition, to my knowledge, ciliary vesicles have not been described on Drosophila EM images, thus parts of Figure 7 are speculative.

Thanks for corrections, we revised as suggested by the reviewer.

Our working model is based on the consensual concept about ciliogenesis. Currently, it is generally believed that ciliary vesicles are involved in the formation of all cilia. Ciliary vesicles are observed at the cilia base in *C. elegans* and mammals. For *Drosophila*, ciliary vesicles are also observed at the cilia base in sensory neurons (Please refer to Fig. 5B in *Vieillard et al., J. Cell Biol. 2016; 214 (7), 7875–889*; and Fig. 1A in *Jana et al., 2018; Nat Cell Biol. 2018;20(8):928-41*).

At many occurrences, the conclusions appear to be overstated:

Page 8, end of second paragraph :

« Probably its N-terminus alone » this is likely overstated. It shows that the N-terminus is required but not sufficient as the C-terminus truncated Cep290mecH mutants do have a TZ gating defects (Basiri et al., 2014).

We removed it.

Page 13: "DZIP1 did not affect the initial docking of the BB": same comment as above for the cep290 mutant. What are the arguments to say that the BB are docked and not only apposed to the plasma membrane?

We appreciate the reviewer to raising this question. Technically, it is difficult to differentiate whether the BB is docked or just apposed to the plasma membrane. We deleted the phrase “did not affect the initial docking of the BB”.

Page 20: second paragraph: "suggests that they have synergistic roles in early ciliary membrane formation". The manuscript only shows that MKS1 is affected not that there are defects in early ciliary membrane formation.

We removed this sentence and revised this paragraph.

Last paragraph of the result section: the last sentence is highly speculative and should be removed.

Removed as suggested by the reviewer.

Some of the results obtained for dzip1 mutant confirm recently published results by Lapart et al, 2019. This should be stated in the result or discussion section.

We revised the manuscript accordingly.

As well, some observations of the cep290 mutants confirm previous published results by Basiri et al., Jana et al. and Lapart et al., and this should be recalled accordingly in the text.

We revised the manuscript accordingly.

Page 21 "regulating DZIP1 mediated early ciliary membrane": incomplete sentence.

Revised. Should be “regulating DZIP1 mediated early ciliary membrane formation”.

**Reviewer #3:**

In this manuscript, Wu et al. report that the N-terminal region of CEP290 plays an essential role in the initiation of transition zone assembly of the cilia in in neurons and spermatocyte of Drosophila. They demonstrate that the N-terminus of CEP290 directly interacts with DZIP1 and recruits DZIP1 to transition zone, where it recruits CBY and RAB8 to assemble the ciliary membrane. The authors propose that CEP290 coordinates the early ciliary membrane formation and transition zone assembly.

Overall, this work is of importance to the ciliogenesis field because little is known about the regulation of early ciliary assembly events. Requirements for CEP290 in transition zone assembly and function has been investigated in several model systems including Drosophila (Jana et al 2018, Li et al 2016). Importantly, CEP290 has previosuly been reported to act upstream of DZIP1 recruitment - which was not mentioned in this paper. Moreover, associations with Rab8 function have been reported and were for the most part noted in this manuscript with exception a paper in 2008 by Kim et al. Thus, the main findings from this work largely relate to the identification of interactions for CEP290-DZIP1 and DZIP1-CBY/Rab8. However, the interaction between DZIP1 and Rab8 and CBY seem rather weak compared to the input.

The novel and critical findings of our study are highlighted as following:

1. We demonstrated that CEP290 is essential for the initiation of ciliogenesis and TZ assembly. This result has not been reported before. Previous reports about CEP290 mainly focused on its role in the hierarchical assembly of TZ core proteins and the formation of Y-link structures.
2. We showed that CEP290 directly interacts with DZIP1, and regulates ciliogenesis initiation and TZ assembly via DZIP1. Although *Lapart et al.* reported that DZIP1 is mislocalized in *cep290* mutants (*Lapart et al. eLife 2019;8:e49307*), but their molecular relationship has not been explored.
3. We showed that DZIP1’s binding factors, CBY and RAB8, jointly regulate ciliogenesis. *dzip1* mutants have much more severe defective phenotypes than *cby* or *rab8* mutants alone, suggesting that defects caused by deletion of DZIP1 cannot be attributed to either CBY or Rab8 pathway specifically.

Both the interaction between DZIP1 and Rab8 and the interaction between DZIP1 and CBY have been confirmed by GST pull down assay and Co-immunoprecipitation assay, and these experiments have been repeated 3 times. Although interactions may be weak, we are confident that they do interact with each other. In addition, The interaction between DZIP1 and CBY in *Drosophila* has been independently reported by *Lapart et al*. (*Lapart et al. eLife 2019;8:e49307*), and the interaction between DZIP1 and Rab8 in mammals has been reported previously by *Zhang et al.* (*PLoS Biol 2015; 13:e1002129*).

Another concern I have is that conclusions drawn about TZ structure and protein localization may be over interpreted from the provided SIM imaging. The quality/resolution of these images seems lower than what has been published by others for the TZ in Drosophila.

Our new result about TZ structure and protein localization is that there is a gap between transition fiber protein FBF1 and TZ core protein MKS1 in auditory cilia, and DZIP1 occupies the gap region between FBF1 and MKS1, and further extended distally, partially colocalizing with MKS1 (as clearly demonstrated in Fig 4E). We are very confident about this observation. Please see our response to your additional question #6.

Additional concerns/questions/suggestions are listed below.

1. The C-terminus of CEP290 has microtubule binding domain while the N-terminus has membrane binding motif. Here the authors show that the N-terminus alone localizes to the transition zone (Fig. 1). How might this fragment be anchored at the transition zone and basal body? Understanding this question could help enhance the mechanistic understanding of this work.

This is a great question. Currently, we do not have a definite insight. It is possible that there is an unknown protein interacting with CEP290 via its N terminus. It will be interesting to investigate its molecular identity in future.

2. The 21A6 antibody recognizes two domains longitudinally in auditory neurons. Which domain is shown in Fig. 1B, 4F and 5B? What are the directions of the cilia in these figures? Showing un-cropped images may help to predict the position of the cilia in these cells.

The domain around the ciliary base domain is shown in all our figures in old version. As the reviewer suggested, we have now shown two domains in the revised version.

3. What are the stages of the spermatocyte in Fig 2C? What structure is the elongated UNC signal related to when the ciliary bud formation is blocked in CEP290 null cells as shown in Fig. 2C and 2F?

They are late spermatocytes.

In late spermatocytes of *cep290* cells, since there is to no ciliary cap, microtubules of the basal body/centriole are abnormally extended. The elongated UNC signal is the UNC protein that localizes to the elongated microtubules.

4. The localization of CG6652 in cep290 null cells is very different comparing to wt and delta C cells in Fig. 2D. The authors claim that this is abnormally extended axoneme. But the distribution of CG6653 does not look like an axoneme. Electron microscopy analysis would be necessary to confirm this structure.

Aberrant extension of microtubules from the basal body/centriole were first described by Prof. Durand lab, please refer to prior EM evidence in Fig. 7A of their paper (*Vieillard et al., J Cell Biology 2016*). The abnormally extended microtubules are not stable, since they often branch and collapse easily.

5. The association between basal bodies and plasma membrane was not defective in later spermatocytes and spermatids in CEP290 dC mutant cells (Fig. 2E). The transition zone can be assembled too (Fig. 2C). Are the cilia buds formed in these cells comparing to wt and null cells as in Fig. 2F and 2G?

Please refer to Fig. 5D of Reference 24 (*Basiri et al., Curr Biol 2014; 24: 2622-2631*), Previous EM images showed that the primary cilium/ciliary cap formed in *cep290^mecH^* mutants. So, the cilia buds should be formed in equivalent *cep290^ΔC^* mutant.

6. The authors indicate that DZIP1 occupied the gap region between FBF1 and MKS1 in Fig. 4E and S3E. However, there is still a gap between DZIP1 and FBF1 in Fig. 4D auditory neurons. Are these representative images?

Sorry for our negligence. This is not a representative image. In this image, the signal of DZIP1 in proximal TZ is less intense, whereas its signal in distal TZ is abundant. If increasing brightness as image showing above, there is no apparent gap between DZIP1 and FBF1. We replaced this image with a representative one.

7. It would be helpful to note in the figure legends that some of the blots are antibody staining while other are Poncuau S. stained for clarity.

Thanks for the suggestion. We included corresponding labels in the figures.

8. Showing images of the whole auditory and olfactory systems can help confirm signal from background. The cropped area in images in Fig 1BCD, Fig.3C, Fig. 4F and Fig. 5AB are too small.

Due to the space limitation, we only showed the representative images of the basal body of a cilium. All signals are homogenous between cilia, and the un-cropped images were provided in Supplementary Fig. 8

**Reviewer #4:**

**Major points:**

1.	Accessibility: Many of the figures are too complex- it is very difficult to see details/labels even at their current full page size (see all Figure 2, Figure 3C graphs, Fig4D,E graphs, Fig 7). Also as most of the panels have only two dyes/fluorophores- I would strongly urge the authors to consider a color blind palette (magenta/green, cyan/orange- see - https://imagej.nih.gov/ij/docs/guide/146-9.html- about 10% of your male audience may be colorblind). Also the dark blue/indigo of the CellMask in 2E, 4H-I and 5F is difficult to discern from the black background- consider magenta or something bright.

Due to the limited space to show a lot of data, we reduced the size of pictures and the font size of labels in some figures. We are sorry for any inconvenience caused by this.

We have rearranged some figures and increased font size, particularly in Fig. 2, Fig. 3C, Fig. 4D, E and Fig. 7.

As suggested, we replaced the dark blue of the CellMask into Magenta in all figures.

For replacing Red with Magenta, we will follow the editor’s instruction later.

2. Allelism: This current study does not address any about protein levels between lines- but puts it down to differences in activities of different protein domains- which should remain an interpretation of the results, given it could be down to just protein levels. There is a big point made about the previously published fly line (Basiri et al 2014; cep290mecH) which causes a premature termination codon resulting from a frameshift, in the 5th last exon of a very large gene- not being a null allele and instead being a milder loss-of-function allele due to remaining action of the C-terminally truncated protein (lacking the final~600 aa). Showing my mammalian bias, one would think this would subject the transcript to nonsense mediated decay, instead of protein truncation. Although speculated this truncated protein species exists, it is never investigated. I know that antibodies are difficult for Drosophila studies but some RNA/semi-quantitative PCR to look at levels and altered splice variants could lead credence to this-

*Drosophila* *cep290* gene encodes a protein of 1978 amino acids. In our *cep290^1^* (c.469-1174Del) mutant, the deletion results in a frameshift and causes a premature stop codon, only the N-terminal 156 amino acids are retained. In our *cep290^ΔC^* (c.4156delG) mutant, the one bp deletion causes a premature stop codon and the C-terminal ((aa 1386-end) loss.

To determine if a C-terminal truncated protein do exist in our *cep290^ΔC^* mutant, we generated an anti-CEP290 antibody against CEP290 N-terminal (292-541 aa). The antibody works well in immunofluorescence assay. By using this antibody, we observed that CEP290 was completely lost in *cep290^1^* mutant, but it still existed in *cep290^ΔC^* mutants (Supplementary Fig. 1D), supporting that CEP290 N-terminal do exist in *cep290^ΔC^* mutant. Unfortunately, the antibody doesn’t work in the western blot assay, we can’t use this antibody to determine if CEP290 is precisely truncated in *cep290^ΔC^* mutant. Notably, the signal intensity of CEP290 was reduced in *cep290^ΔC^* mutant compared to WT.

In addition, as the reviewer suggested, we also checked the transcription level and alternative splice variants in *cep290^ΔC^* mutant. The CDS of *Drosophila cep290* gene is 5937 bp long. As the full length CDS is too large to amplify. To determine if there are alternative splice variants in *cep290^ΔC^* mutant, we amplified the C-terminal half of *cep290* CDS (2662-5937) in *cep290^ΔC^* mutant, no alternative splice forms were found (Supplementary Fig. 1B). Furthermore, amplifying a shorter C-terminal fragment showed that the transcription level of CDS was indeed reduced in *cep290^ΔC^* mutant, probably due to nonsense-mediated mRNA decay. Together with the finding that CEP290 protein is reduced in *cep290^ΔC^* mutant, our result is consistent with the reviewer’s speculation.

and that the cep290l and cep290ΔC lines generated here by genome editing, where larger on-target 'off-target' effects like inversions or larger deletions, make a phenotype seem more severe.

To eliminate the possible “off-target”, before phenotypic analyses, every mutant fly strain was outcrossed 3 times to dilute any potential off targeted lesion.

Similarly, the transgenic over-expression some of the GFP CEP290 variants result in defective motility where the N-terminal CEP290 'may play a dominant negative role'- if this was true then heterozygous carriers for the cep290ΔC allele would have a similar uncoordinated, motor phenotype which they do not. Indeed according to the Basiri paper 'cep290mecH homozygotes (cep290) and hemizygotes (cep290/−) cannot stand….cep290mecH heterozygotes (cep290/+) and rescue (Cep290 and Cep290-GFP) are normal.' The lines of argument here are contorted.

Thank the reviewer for raising this question. One possible explanation is that the expression level of N-terminal fragment is related to the severity of phenotype.

We removed the dominant negative statement as suggested by the reviewer in Minor points #9.

A safer approach would be like the Lampard paper uses an enhancer trap line- and just says it is a strong hypomorph and has a similar phenotype- loss of FAM92 and DZIP from the transition complexes, and '76% of aberrant axonemal growth, suggesting that basal body to membrane attachment is compromised in this mutant'. Genetics is complicated and sometime a rabbit hole not worth completely going down to prove your alleles are in fact nulls. Indeed in the clinical setting, we know that generally the severity of CEP290-associated phenotypes seems to correlate with the residual amount of functional protein that a mutant allele could produce (Drivas et al., 2015; Shimada et al., 2017)- something you have not looked at here rather tie it to domains. This is a weakness for me.

We appreciate the suggestion and revise the manuscript accordingly to be more accurate.

**Minor points:**

1. Awkward and inexact: Abstract 'Cilia play critical roles in organ generation and maintenance' consider 'during embryonic development and adult homeostasis'.

Revised as suggested, thanks.

2. Introduction, p3, first line- typo '…that extend from the surface of most eukaryotic…'.

Corrected.

3. Introduction, p4, second line- typo '…which connect proximal axonemal…'.

Corrected.

4. Introduction, p4, second line Long sentence consider splitting- 'to the ciliary case membrane. These have been proposed to organize… on the membrane surface. The transition zone functions…'.

Thanks for the suggestion. The sentence has been divided into two sentences.

5. Introduction, p4. Second paragraph, third line. Italicize gene 'CEP290' and consider rewording from list of disorders to a more informative statement for non-expert readers like '…several ciliopathies ranging from non-syndromic retinal degeneration (i.e. Leber Congenital Amaurosis (LCA) to syndromic disorders including Senior-Loken syndrome (SLS),…'

Revised as the reviewer’s suggestion.

6. Introduction, p4. Second paragraph, missing text. '…diseases and phenotypes associated with CEP290 mutations highlight the multiple and critical…'. Gene names should always be italics.

Revised as suggested. Thanks!

7. Introduction, p.5- inconsistency. I may be missing something but the statement 'We proposed that ciliary membrane assembly is a prerequisite for TZ assembly initiation' seems to be not what it is shown in your summary figure 7- where binding of CEP290 to apical membrane and recruitment of TZ facilitators DZIP and CBY occur before recruitment of Rab8/ciliary membrane vesicles. To the model is the inverse of this 'We proposed that TZ assembly initiation is a prerequisite for ciliary membrane assembly…'

We appreciate the reviewer for raising this question.

In general, TZ refers to the Y-linked structure aera composed of TZ core components MKS module and NPHP module. DZIP1 and CBY are required for TZ assembly, but they are not TZ core components. Here, our results suggest that DZIP1 and CBY control TZ assembly by regulating ciliary membrane formation. What we mean was that “ciliary membrane assembly is a perquisite for TZ core structure assembly initiation”.

To be more accurate, we removed the word “initiation” from this sentence in the revision.

8. Results, p.5, last paragraph, missing text. '…microtubules and ciliary membranes, where its C-terminus…'. Consider referring to Figure 1A here.

Revised as suggested.

9. Results, p.5/6- clarity. Consider emphasizing constructs are overexpressed in wild type flies. Consider removing dominant negative statement at end of paragraph (see above).

Revised as suggested.

10. Figure 1 A-D- do you know that all these GFP constructs are expressed- the lack of staining at the basal body of cilia for CEP290-M could simply be due to unstable protein. Also- I am perplexed: Figure 1B vs 1E- why such a different look to the cilia localizations between modalities- the confocal has two closely aligned structures and the SIM data only has one a field of view that is only double the magnification of the first, according to scale bars. Maybe a statement clarifying this is needed in the legends.

For CEP290-M-GFP, we saw GFP signals in cytosol, so it should be expressed.

Cilia in Figure 1B and 1E comes from the Johnston’s organ (JO) of *Drosophila*. JO is a specialized Chordotonal organ (ChO) which is composed of a various number of scolopidium. A scolopidium usually consists of two neurons with cilia reside on their dendrite tips. In Figure 1B, the two closely aligned structures are the TZ of two cilia in a scolopidium. In Figure 1E of our old version, only one cilium in a scolopidium was showed. Notably, the distance between the two cilia in a scolopidium may look different, depending on the viewing angel.

We have replaced Fig.1E with new images showing two cilia in a scolopidium.

11. Results, p.7, for transparence- add reference to previously published alleles for cep290. 'Similar to previously reported alleles, both cep2901 and cep290ΔC were uncoordinated….. (Basiri et al 2014, Lampard et al 2019).

Revised.

12. Figure 2A- for clarity, consider annotating where cep290mecH falls. Also explicitly add into the figure legends that these are predicted protein sizes- both of your CRIPSR alleles (and cep290mecH) may be subject to NMD and thus have little protein of any size to speak of.

Revised as the review’s suggestion.

13. Results, p.9, last paragraph- clarify. '…leading to loss of cilia/TZ' as the paragraph is written these structures never form- consider 'completely blocked, such that cilia with transition zones never form'. The following sentence may need some gentle reworking for non-fly enthusiasts, '…resulting in subsequent abnormal elongation of membraneless ciliary axonemes within the cytoplasm'.

Revised as suggested.

14. Results, p.10, typo, first sentence. '…the initiation of TZ assembly, spermatogenesis….'.

Revised.

15. Results, p.11, typo, second sentence. '…intensities were severely reduced compared to….'.

Revised.

16. Results, p.11, emphasis. Tone down final statement first paragraph- ' …in sensory neurons, the CEP290 N-terminus may be essential for initial TZ assembly, whereas TZ elongation or maturation may involve a tissue-specific requirement for the C-terminus of CEP290.'

We appreciate the suggestion. Revised.

17. Results, p.11, typo. '… we employed a yeast two hybrid….' Also- 'Drosophila DZIP1 is the ortholog of mammalian DZIP1..'

Revised. Thanks!

18. Results, p.11, clarification. What is the CEP290-ΔN construct? What is in it is never detailed anywhere and is confusing with what is outlined in figure 1A.

We labeled in figures and indicated in legends.

19. Results, p.12, clarification. 'While the role of Drosophila DZIP1 in ciliogenesis was unknown when we started this project, a very recent publication has confirmed our findings (Lampard et al 2019).'

Revised as suggested.

20. Results, p.13, typo. '…completely lost but CEP290 persisted…'. Also 'Furthermore, similar to cep2901 mutants…'

Revised.

21. Results, p.14, typo. '… C-terminus may have tissue-specific roles in regulating…'.

Revised.

22. Results, p.17, typo. '…compensate for the loss of Rab8, as has been reported for Rab8 and Rab10 in mammalian ciliogenesis…'.

Revised as suggested.

23. Results, p.17, clarify. I was confused by the statement '…then facilitate the Rab8- and its redundant player- mediated…'. Consider '…then facilitate the Rab8- and its yet-unknown compensatory mechanism regulating…'.

We have deleted this sentence as suggested by another reviewer.

24. Discussion, p.18, see point 7. '…TZ assembly is completely blocked in dzip1 mutants suggests that early ciliary membrane formation is a prerequisite for TZ assembly initiation'. Again I think this is the opposite of what your data shows- TZ assembly predates early ciliary membrane formation in your analysis.

We deleted the word “initiation”. Please refer to our answer to question #7.

25. Discussion, p.18, careful. The discussion about CEP290 mutations in human patients- there are retinal specific isoforms for many key RP genes which are believed to be the reason for the syndromic versus non-syndromic divide for some of these clinical phenotypes. This should be referenced. Again although the genotype-phenotype correlation is challenging, in general it is believed that the severity of CEP290-associated clinical phenotypes appears to reflect the residual amount of functional protein produced by a mutant allele (Drivas et al., 2015; Shimada et al., 2017). This current study does not address any of this- but puts it down to protein domains- which should remain an interpretation of the results, given it could be down to just protein levels.

We appreciate the suggestion and rewrote this paragraph.

We agree with the reviewer that protein level is related to the phenotype. We also think that both the level of protein expression and its domain are important to its function.

26. Discussion, p.19, typo. 'In Drosophila, the centriole…'. Also take out the '(our unpublished data)' , either you show it or don't mention it at all.

Revised as suggested. The sentence of “our unpublished data” was removed.

27. Discussion, p.20, typo. '…understand the mechanisms of…'.

Revised.

28. Discussion, p.20, clarify (see point 23). Change '…then facilitate the Rab8- and its redundant player- mediated…'. Consider '…then facilitate the Rab8- and its yet-unknown compensatory mechanism regulating…'.

Revised as suggested.

29. Discussion, p.20, nomenclature. Mouse alleles should be title case italics- Dzip1 or Dzip1l. See final paragraph too. There is also a typo here- mice is plural so should be 'have much more severe…'. Same for Cby and Fam92a. Within species, they are not orthologs, they are paralogs. Final sentence in paragraph change to '…the existence of so many paralogs complicates the study of their genetic relationship'.

Revised. Thank you so much!

30. Discussion, p.21, missing word. '….regulating DZIP1-mediated early ciliary membrane WHAT?….'.

Sorry for our writing. Should be “regulating DZIP1-mediated early ciliary membrane formation”

31. Methods, p.24, add information. The microscopy and image analysis- how was the quantitative imaging done- how many flies, how many basal bodies/TZ to get traces'.

Corresponding information has been added to this method section.

Information about “n” number was also indicated in figure legends.

32. Acknowledgements, p.35, typo. Change to '…for their technical support'.

Revised.

33. Figure Legends, 4 title, clarify. '…with the N-terminus of CEP290, and dzip1ΔC deletion mutants…'

Revised as suggested.

34. Supplementary Figure legends, p.42. Clarify PCR is of what- genomic DNA? In figure S1E- there is a typo throughout with your driver- should be elav-GAL4 not ealv. Figure S1- add in what acronym DBB and PBB mean.

All revised. Thanks.

35. Supplementary Figure legend 2, p.43. There is a typo in the figure for H. Sapiens several times.

We are sorry for our mistakes. Revised.

36. Supplementary Figure legend 3, p.44. Typo '…to the tip of the flagella.'

Revised.
