# Supplementary material for: CEP290 is essential for the initiation of ciliary transition zone assembly
Source: PLoS Biol. 2020 Dec 28;18(12):e3001034. doi: 10.1371/journal.pbio.3001034 (PMC7793253; doi:10.1371/journal.pbio.3001034)
Supplement: Attachment — Submitted filename: Response to Reviewers.docx [file pbio.3001034.s013.docx]

**Rev. 1:**

In this revised manuscript, Wu et al. largely address the concerns I raised previously. While it is unfortunate that the Cep290-Dzip1l interaction could not be dissected more precisely, the authors made a reasonable effort, and they revised the text accordingly. I would encourage the authors to include the coIP showing interaction of mammalian Dzip1l and Cep290 if possible. There are also some grammatical errors that should be corrected before publication, and parts of the Discussion are not as clearly written as the rest of the manuscript. These minor issues notwithstanding, I believe the manuscript is suitable for publication in PLoS Biology.

We thank the reviewer’s helpful comments and appreciate for his/her supporting our work for publication.

As the reviewer suggested, we have included the Co-IP data showing the interaction of mammalian CEP290 and DZIP1L in S7 Fig. We have also checked for grammatical errors and typos, and revised the manuscript accordingly.

**Rev. 2:**

The authors have answered to all my initial requests and the additional data reinforce their conclusions.

In particular they have corrected all the overstatements and added interesting data showing functional interactions between Fam92 and Rab8, indicating that Rab8 and Fam92 or Cby cooperate during spermatogenesis.

Thanks! We thank again the reviewer for all the helpful comments.

Minor point:

Page 13 fourth line: MKS6 is not presented on sup Fig 4C.

Thank you for reminding us, we have included the image of MKS6 in S4C Fig during the revision.

page 14 last paragraph: "is still existed" check English grammar.

Sorry for our mistake. Revised.

**Rev. 3:**

The revised manuscript has been improved and addressed most of my concerns. Though how the N-terminus of CEP290 is anchored to the basal body to recruit Dzip1 is not completely clear. Overall this work and model proposed for CEP290-Dzip1-Rab8 pathway in TZ assembly initiation is important to the field.

Thanks! We thank the reviewer for appreciation of our work.

**Rev. 4:**

The revised work by Wu et al represents a significant genetic and biochemical study into early stages of ciliogenesis in Drosophila regulated by CEP290 through the DZIP1-CBY/Rab8 module. Using a range of imaging modalities and generating many genetic tools, the work is novel and sound- appropriate for publication in a revised manner in PLoS Biology.

We thank the reviewer for all his/her valuable comments and suggestions. Please see our point-by-point response to the remaining concerns.

1. Accessibility: I am disappointed the authors did not choose to make their data more accessible to the broad readership of the journal. 10% of all readers, including some of the corresponding authors whose work they cite here, are red-green colorblind and as such are unable to interpret the bulk of the data presented in this manuscript. Changing pseudocolors in two color panels is very easy in FIJI with plug-ins- it would take the authors a half-day tops but would be so inclusive, improving pick up and citation of their work. (On a side note, I do wish PLoS would make this policy and mandate, not be optional).

We thank the reviewer’s helpful suggestion and apologize for not changing Red to Magenta in our first revision.

In this revision, we have changed Red to Magenta in all images.

2. Allelism and expression: as I wrote, we know from human genetics that levels of variant expression are key for CEP290 phenotypes- this is ultimately at the protein level, which the authors are unable to look at. I think this statement needs to be made explicitly as a caveat to their interpretation in the discussion. Something akin to 'Without a robust antibody to survey expression to validate levels of CEP290 protein expression within our alleles, we cannot fully unpick how much of our phenotypes are due solely to reduced levels versus requirement for specific domains. We suspect it is likely a combination of both'.

We appreciate the reviewer’s suggestion. This sentence has been appended to the last paragraph on Page 11.

We agree with the reviewer that without checking protein level, the phenotypic difference between *cep290^1^* and *cep290^ΔC^* cannot be attributed to the N-terminal domain alone. Therefore, in our first revision, we have revised and toned down the function of “N-terminal of CEP290”. Such as, we delete “the N-terminus of” from the sentence “the N-terminus of CEP290 plays an essential role in the initiation of TZ assembly”, changed this sentence to “CEP290 plays an essential role in the initiation of TZ assembly”.

Reviewer 1 (Question 2) also raises issues of protein levels, as does reviewer 2 who wrote ‘…all the experiments based on the expression of these truncated forms, could there be an effect of the expression levels?’ Their response ‘To ensure that CEP290 and its truncations were expressed at a comparable level, all these transgenes were inserted o the same integration platform (the attP site of 25C6 locus on chromosome 2), and they were driven by the same ubiquitin promoter. Therefore, it is unlikely that the different defective phenotypes with expressing various CEP290 truncations are due to their different expression levels’. Again, this reflect only transcript level and not stability of the protein- this is emphasized in Supp Fig 8 where there is no GFP expressed for CEP290-M suggesting it is not stable. Without a GFP blot to show similar levels of protein expression, untangling protein instability/levels versus changes in localization cannot be unpicked.

Thanks for correcting us. Reviewer #2 raised this question about our statement of dominant negative effect of expressing CEP290-N in our original version. Since we cannot rule out the possibility of expression levels effect, this strong statement has been removed in our first revision.

In addition, we agree with the reviewer that western blot is needed to detect the protein level of different truncations. Therefore, we have performed the western blot assay, and found that the protein level of CEP290-N and CEP290-M were similar, but the protein blot level of CEP290-C was the lowest. Since CEP290-C-GFP demonstrated a strong immunofluorescene signal on TZ despite appearing relatively weak on protein blot, we feel unconvinced to draw conclusions on the effect of the expression level by western blot alone.

Again, in the revised version, we removed the dominant negative effect statement, revised and toned down functions of specific domains.

**Minor points:**

1. A comment: My point about was about on-target 'off-targets' in their crispant alleles- larger or complex rearrangements at the locus that because they are at the locus and cannot be 'eliminated by outcrossing 3 times to dilute any potential off targeted lesion'. Large long read sequencing is necessary and/or southern blots to really confirm, but I suspect were not done.

We agree with the reviewer that outcrossing cannot absolutely eliminate off-target mutations in the region adjacent to the gene of interest, long-read sequencing the region adjacent to the gene of interest is a way to exclude this kind of off-targets.

Of note, using genetic rescue to analyze phenotypes is another powerful way to confirm the relationship between phenotypes and genotypes. Although we did not sequence the region adjacent to our gene of interest, our genetic rescue experiments have demonstrated a strict CEP290 specific role in our phenotypic analysis.

2. Last paragraph of the introduction- it is all past tense, but work you describe in the paper- present tense is more apropos.

Thanks for the suggestion. Revised.

3. Figure 1C legend: Sequencing of what gDNA or cDNA?

The legend of S1C Fig has been revised to “cDNA sequencing verification of c*ep290^ΔC^* mutant.”

Also in this figure why are the numbers different at the bottom of the chromatograms- consider removing as they are confusing, without adding value.

Removed as suggested.

4. p7 'This is consistent with the transcription level of cep290 in cep290ΔC mutant' should read ' consistent with the transcript level'- you are assaying only transcript level (result of production and turnover).

Revised.

5. P8 Protein DILA should be all caps.

Revised.

6. Top of p12 typo: 'Drosophila DIZP1 is' should read 'Drosophila DZIP1 is'

Revised.

7. Page 12 Overexpression in a cell line does not constitute in vivo 'GFP-tagged CEP290 and HA-tagged DZIP1 in cultured Drosophila S2 cells'- removing 'also occurs in vivo,' without losing emphasis.

Removed as suggested.

8. Top of p16- typo 'cby or fam92 single mutants'

Revised.

9. Top of p18- typo single or plural- clarify 'Then we created a fam92 deletion mutants'

Change to “Then we created *fam92* deletion mutants”.
